# Epigenetic Patterns and Geographical Parthenogenesis in the Alpine Plant Species *Ranunculus kuepferi* (Ranunculaceae)

**DOI:** 10.3390/ijms21093318

**Published:** 2020-05-07

**Authors:** Christoph C. F. Schinkel, Eleni Syngelaki, Bernhard Kirchheimer, Stefan Dullinger, Simone Klatt, Elvira Hörandl

**Affiliations:** 1 Department of Systematics, Biodiversity and Evolution of Plants (with Herbarium), University of Goettingen, Untere Karspüle 2, 37073 Göttingen, Germany; eleni.syngelaki@uni-goettingen.de; 2 Department of Botany and Biodiversity Research, University of Vienna, Rennweg 14, 1030 Vienna, Austria; bernhard.kirchheimer@univie.ac.at (B.K.); stefan.dullinger@univie.ac.at (S.D.); 3 Section Safety and Environmental Protection, University of Goettingen, Humboldtallee 15, 37073 Göttingen, Germany; simone.klatt@zvw.uni-goettingen.de

**Keywords:** *Ranunculus kuepferi*, apomixis, geographical parthenogenesis, methylation-sensitive AFLPs, polyploidy

## Abstract

Polyploidization and the shift to apomictic reproduction are connected to changes in DNA cytosine-methylation. Cytosine-methylation is further sensitive to environmental conditions. We, therefore, hypothesize that DNA methylation patterns would differentiate within species with geographical parthenogenesis, i.e., when diploid sexual and polyploid apomictic populations exhibit different spatial distributions. On natural populations of the alpine plant *Ranunculus kuepferi*, we tested differences in methylation patterns across two cytotypes (diploid, tetraploid) and three reproduction modes (sexual, mixed, apomictic), and their correlation to environmental data and geographical distributions. We used methylation-sensitive amplified fragment-length polymorphism (methylation-sensitive AFLPs) and scored three types of epiloci. Methylation patterns differed independently between cytotypes versus modes of reproduction and separated three distinct combined groups (2x sexual + mixed, 4x mixed, and 4x apomictic), with differentiation of 4x apomicts in all epiloci. We found no global spatial autocorrelation, but instead correlations to elevation and temperature gradients in 22 and 36 epiloci, respectively. Results suggest that methylation patterns in *R. kuepferi* were altered by cold conditions during postglacial recolonization of the Alps, and by the concomitant shift to facultative apomixis, and by polyploidization. Obligate apomictic tetraploids at the highest elevations established a distinct methylation profile. Methylation patterns reflect an ecological gradient rather than the geographical differentiation.

## 1. Introduction

Epigenetic processes are regulatory mechanisms that may affect phenotypes without altering DNA sequences [1,2,3]. They undergo constant transformation [4] and may cause high phenotypic plasticity and potentially heritable variation [5,6,7]. Among others, methylation at the 5′ carbon of cytosine is an epigenetic mechanism, and it is particularly important in plants [4,8]. It affects individual development through control of gene regulation and expression, as well as cell differentiation by activating, reducing or completely disabling the activity of particular genomic segments [9,10,11]. Multiple other studies have demonstrated the effects of methylation patterns on ecologically relevant traits and trait plasticity [12,13]. 

Genomic rearrangement due to hybridization or polyploidization itself can induce changes in DNA methylation patterns [9,14,15,16,17] as well as environmental stresses [13,18,19,20,21,22]. In plants, temperature seems to trigger certain methylation conversions [23,24,25]. Such sensitivity might be associated with high plasticity in response to extreme environments [20], especially if induced methylation changes are heritable [19,26].

Importantly, in our context, cytosine methylation is also involved in the complex regulatory mechanisms for expression of apomictic versus sexual reproduction [27,28,29,30,31,32,33]. Apomixis is defined as a form of reproduction that produces asexually developed seeds as a heritable trait [34,35,36,37]. Apomixis per se is not directly dependent on polyploidy, as the trait occurs in many diploid plants. Only gametophytic apomixis, the type relevant for our study, is more frequent in polyploids [38]. Gametophytic apomixis involves unreduced embryo sac formation from either somatic nucellus cells or via restitutional meiosis during megaspore development [35,37]. Apomictic seeds sustain the genome configuration of their parental progenitors and hence establish the fixation of any genotype. Although apomixis is widespread in natural populations and occurs among at least 78 families and more than 290 genera of angiosperms [39], genetic and epigenetic control mechanisms on its expression still remain elusive. In general, apomixis appears to be triggered by a spatial (ectopic, i.e., in another cell) or temporal (asynchronous) deregulation of the genes that control the sexual meiotic development of female gametes [27,31,35,40,41]. Almost all natural gametophytic apomictic plants are polyploid [42]. However, recent studies suggest that also this type of apomixis can originate spontaneously in diploids, usually at low frequencies [43,44,45,46]. Hence it is possible that epigenetic change in diploids is involved in the emergence of apomixis, whereas polyploidy just has indirect positive effects on the establishment of asexual lineages [47].

Apomictic polyploids frequently exhibit rapid range expansions which often result in larger distribution areas compared to their diploid progenitors, a pattern called geographical parthenogenesis [48,49,50]. Such range expansions of polyploid apomicts are not typical for sexual polyploids [50]. Apomicts express a tendency to higher latitudes as well as elevations and accordingly to colder climates [45,48,51]. Natural spontaneous origins of apomixis have been evident during climatic fluctuations throughout the Pleistocene [42,47]. Experimental studies affirmed that cold temperature regimes can increase the proportions of asexual reproduction in diploid plants [52]. Exceedingly cold temperatures are further known to cause restitutional meiosis and consequently unreduced gametes [53,54,55,56]. Unreduced female gamete formation is a major component of apomixis and at the same time a pathway to polyploidization [57]. Hence, low temperature could be one of the natural triggers for shifts to apomixis [47,52]. However, it is so far unknown whether methylation patterns differ according to the main factors polyploidy, apomixis and temperature regimes in natural populations. 

To improve our understanding of the relationships between ploidy, reproduction mode, extreme environments and methylation patterns, we studied natural diploid and tetraploid populations of the alpine perennial species *Ranunculus kuepferi* Greuter and Burdet along an elevational gradient. This species is a model system for geographical parthenogenesis [58,59,60,61], with diploid populations confined to the southwestern European Alps, while tetraploids inhabit previously glaciated areas of higher elevations in the central and eastern Alps, the Apennines, and on Corsica (maps in References [45,58,61,62]). Triploids occur only in a small sympatric contact zone of diploid and tetraploid populations [58]. Tetraploid populations furthermore exhibit a pronounced niche shift towards colder temperatures in the Alps [45,60,61]. Diploid individuals reproduce sexually, with few exceptions of spontaneously generated apomictic seeds, while tetraploid plants are facultative apomictic with varying proportions of sexual and asexual seed formation [45]. Apomictic development starts ectopically from a somatic cell, a pathway called apospory [62]. Combination effects of niche shifts and better founder abilities of apomictic plants explain the larger distribution area of tetraploids in the Alps [61]. 

Previous population genetic studies would not support a hypothesis that genetic differentiation would explain the geographical pattern of cytotypes [63]. These authors had shown with AFLPs and microsatellite studies that tetraploid populations of *R. kuepferi* are autopolyploids and share 97% of AFLP fragments with diploids. Comprehensive AFLP-based population genetic studies by Reference [59] over the whole distribution area revealed just for diploids geographical structure and isolation by distance between geographical groups in their glacial refugial areas. Tetraploids, however, showed no spatial structure and homogeneous patterns of just three genetic partitions over the Alps, no isolation by distance and a similar level of genetic variation within and among populations as their diploid progenitor populations [59]. Molecular dating [61] revealed that tetraploids originated just 10,000–80,000 years ago, which fits with the rapid postglacial colonization scenario as suggested by simulations of range expansions [62]. The high genetic homogeneity in apomicts is also congruent with a recent polyploidization event. We hence hypothesized that epigenetic rather than genetic variation might correlate to the observed shifts to apomixis and to a colder climate of the tetraploid cytotype [45,60]. 

Here, we aim to compare methylation patterns between cytotypes and reproduction modes of *R. kuepferi* to evaluate the extent of differentiation according to each of these factors. Previous studies suggested that apomixis and polyploidy had strong combinational effects on range expansion [61], as predicted by theory [50]. Hence, we evaluate diversity and differentiation of methylation patterns of four combined groups (2x sexual, 2x mixed, 4x mixed, 4x apomictic) as they were found in natural populations [45]. Finally, we test hypotheses that methylation patterns would either follow a spatial differentiation or an ecological gradient according to the niche shift of tetraploids [60,61], or a combination of both. Based on correlations of methylation patterns to the mode of reproduction, ploidy level, spatial structure and environmental factors, we tried to improve our understanding of how epigenetic variation contributed to establishing a pattern of geographical parthenogenesis.

## 2. Results

### 2.1. Differentiation of Cytotypes, Mode of Reproduction and of Combined Groups

Methylation-sensitive AFLP genotyping revealed 1088 scorable fragments in total. Separate analyses of reproduction modes and methylation patterns differed within the same ploidy level. One-factorial ANOVA (analysis of variance) indicated significant deviations between cytotypes (*F*(1, 364) = 14.29; *p* < 0.001) and reproduction mode [*F*(2, 363) = 25.31; *p* < 0.001] but not methylation status (*F*(2, 363) = 1.69; *p* < 0.187). AMOVA (analysis of molecular variance) revealed a significant influence of both cytotype (*p* < 0.001) and reproduction mode (*p* < 0.001) on the total epigenetic variances found (Table 1).

In the combined groups, the number of polymorphic fragments was 682, 404, 629 and 419 in 2xS, 2xM, 4xM, and 4xA, respectively. The number of private markers was lowest among 2xM (24) and highest for 4xM (140). The mean Shannon diversity index was lowest within the 4xA group (*H’* = 0.189) and ranged from *H’* = 0.259 to *H’* = 0.278 in the other groups. More pronounced differences were found in the marker type comparison between non-, external- and internally methylated epiloci (Table 2), where 4xA possessed significantly less non-methylated (17.91%, *p* < 0.001) and externally methylated fragments (16.40%, *p* < 0.001), but significantly increased internally methylated (69.91%, *p* < 0.001) markers compared to the three other groups, which is also reflected in the low values for private markers and the Shannon indices (Table 2). The pairwise comparisons of groups with ANOVAs supported these findings (Table 3) and revealed highly significant differences between 4xA and all other groups (*p* < 0.001) among all subsets of epiloci. 

**Figure 1:**
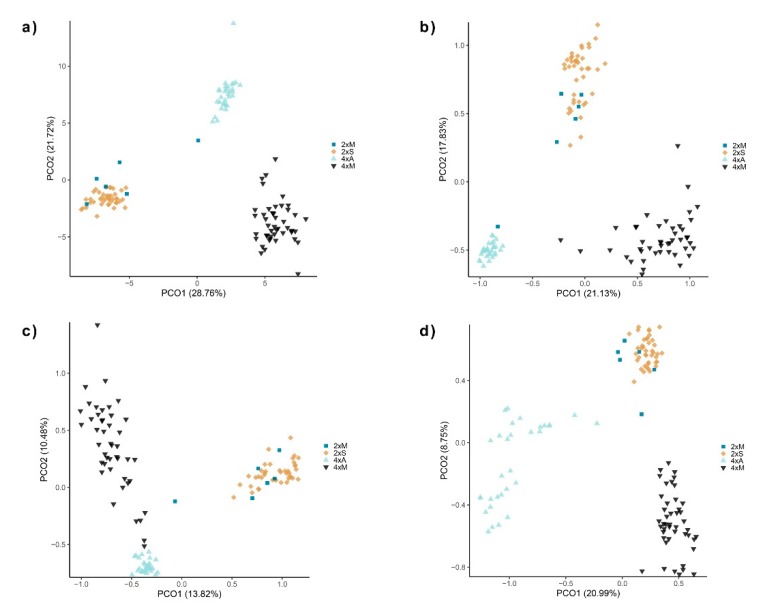
Non-Metrical dimensional scaling (NMDS) plots of 123 individuals of alpine *R. kuepferi* based on the variation of epiloci. Color code represents the predefined combined groups (2x sexual, 2x mixed, 4x mixed, 4x apomictic). (**A**) all epiloci combined, (**B**) nonmethylated epiloci, (**C**) externally methylated epiloci, (**D**) internally methylated epiloci.

Multidimensional scaling diagrams showed pronounced differentiation between three of the four predefined groups (Figure 1). Cytotypes were clearly separated, and within the tetraploids the 4xM and 4xA each formed a distinct cluster. Among diploids the 2xS and 2xM samples clustered together, although one sample of the 2xM group tended to the 4xA cluster. The clustering remained largely the same considering the different epiloci separately. Differences between combined groups occurred in particular in the clustering of internally methylated markers (Figure 1D), with a broader scatter in the 4xA group.

**Figure 2:**
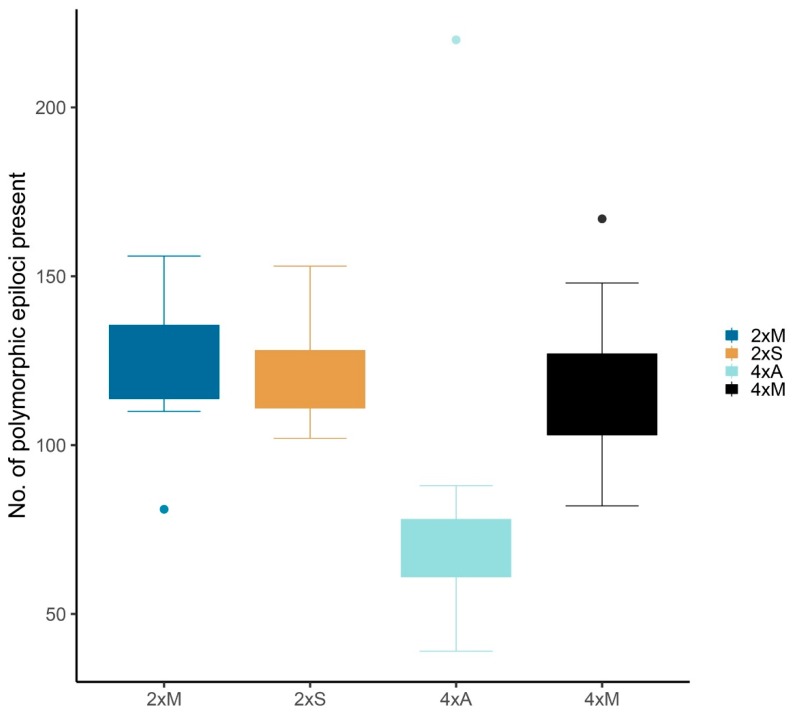
Boxplots of the number of polymorphic epiloci detected in 123 individuals of alpine *R. kuepferi* in the four combined groups (2x sexual, 2x mixed, 4x mixed, 4x apomictic; X-axis) and mode of reproduction (color code). Dots represent outliers.

Boxplots summarizing all epiloci indicated a lower amount of polymorphic epiloci present within the 4xA group compared to the other groups, which exhibited similar frequencies (Figure 2; goodness of fit: Chi-squared = 29.943, df = 3, *p* < 0.001). This is mostly due to a lower amount of non-methylated and externally methylated epiloci (see also Table 2). Pairwise comparisons of combined groups with ANOVAs, where either ploidy (2xS–2xM and 4xM–4xA) or mode of reproduction (2xM–4xM) was kept constant, indicated significant differences (*p* < 0.005) among types of epiloci (Table 3): nonmethylated and internally methylated loci differed significantly between ploidy levels with a mixed mode of reproduction (2xM–4xM). Sexual and mixed reproduction differed in nonmethylated and externally methylated loci in diploids (2xS–2xM), and all three epiloci differed between mixed and apomictic reproduction in tetraploids (4xM–4xA).

AMOVAs further indicated epigenetic differentiation to be higher within combined groups than among them in all comparisons, with percentages among groups ranging from ~15% to 33% in the methylated loci (Supplementary Table S1). F_st_ values for either cytotype or reproduction mode or combined groups as grouping factors were very similar when compared within the same epilocus. Regarding types of markers, F_st_ values were highest for non-methylated loci and lowest for externally methylated epiloci (Supplementary Table S1). Regarding locus-by-locus AMOVA, the overall percentages of significantly differentiated epiloci ranged from 4.17% (2xM) to 32.99% (4xM). Significant differences were found mainly between different reproduction modes (2xS vs. 2xM, 4xM vs. 4xA), between cytotypes (4xM and 2xM) as well as between 2xS and 4xA.

### 2.2. Geographical and Spatial Effects

Comparing epigenetic variances to geographical occurrence of individual plants with a stratified Mantel test revealed no significant geographical structuring, neither among (*r* = 0.155, *p* = 0.864) nor within cytotypes (diploids: *r* = 0.136, *p* = 0.721; tetraploids: *r* = 0.167, *p* = 0.927). This result remained unchanged when Mantel tests were run separately for the subsets of non-, externally- and internally methylated epiloci for both diploids and tetraploids (all *p* values > 0.05). Moran’s *I* values for spatial autocorrelations were low for all groups and methylation status (Table 4), indicating that no global spatial autocorrelation of methylation patterns appeared in the whole study area. In contrast, we observed high values for Geary’s *C* among 18 individuals of 2xS, 14 of 4xM and four of 4xA, respectively. For combined groups, Geary’s *C* ranged from 1.178 to 1.901 in 2xS, 1.033 to 4.401 in 4xM and 1.262 to 1.908 in 4xA, and these values are indicative of negative local spatial autocorrelation. 

### 2.3. Environmental Influences

Spatial autocorrelation (Moran’s *I* values) was correlated both for diploids and tetraploids with all environmental variables tested (elevation, mean annual temperature, and annual precipitation) (Table 5). On the local scale, high values for Geary’s *C* can be found at all elevations and values of mean annual temperature and annual precipitation (Supplementary Table S2). There was some clustering of high *C* values in connection with geographically close populations, but no significant overall correlation with any of the variables or any of the species groups.

Logistic regression revealed significant correlations (R^2^ = 0.89) between epigenetic variation and elevation in 22 epiloci (non-methylated: six; externally: 15; internally: one, Supplementary Figure S1), and between epigenetic variation and mean annual temperature (R^2^ = 0.29) in 36 epiloci (non-methylated: nine; externally: 23; internally: four; Supplementary Figure S2). It is notable that three of each non- and internally-methylated, as well as eight externally methylated epiloci are solely correlated to mean annual temperature (Supplementary Table S3). We could not find candidate loci with significant correlations to annual precipitation.

## 3. Discussion

Our study explored cytosine methylation patterns in di- and tetraploid cytotypes and in different reproduction modes of *R. kuepferi*. We supposed that polyploidy, as well as the mode of seed production, correlates to distinct patterns of epigenetic variation between di- and tetraploids. Previous AFLPs studies indicated that genetic divergence of diploid and tetraploid cytotypes is in this species extremely low (only 3% private fragments in the tetraploids), and genetic variation within and among populations in the two cytotypes is on a similar level [59,63]. In contrast, we found here distinct epigenetic patterns related to cytotype and between modes of reproduction in tetraploids. Additionally, we observed different patterns for non-methylated loci compared to internally and externally methylated epiloci. Hence, we expect that our epigenetic patterns do not just reflect genetic background variation, which is always to some extent underlying methylation variation [22]. We further tested for correlations of methylation patterns to spatial distribution and key climatic variables to understand the effects of the observed distributions and the niche shifts of the tetraploid cytotype [60,61]. In contrast to the genetic pattern in *R. kuepferi*, methylations do not reflect a global geographical pattern but rather an ecological gradient. In other plant species, epigenetic differentiation was better explained by environmental variation than by geographic distance as well [65,66,67]. 

### 3.1. Epigenetic Patterns, Ploidy and Mode of Reproduction

Epigenetic patterns differed in separate correlations to cytotype or mode of reproduction, both in ANOVAs and in AMOVAs. Likewise, F_st_ values in AMOVAs suggested a similar degree of differentiation according to cytotype and mode of reproduction, which strongly supports a hypothesis of the combination effects of these two factors. Accordingly, we focused on further evaluations of combined groups. Here, our NMDS plots of individuals showed a clear distinction of methylation patterns in three main clusters (2xS+2xM, 4xM, 4xA). Of particular interest is the pronounced separation between obligatory apomictic tetraploids and those with mixed reproduction. Samples of diploids with mixed reproduction cluster within the obligate sexual diploids in all epiloci, probably due to the very small sample size of the mixed reproducing diploids, and also due to their geographical occurrence within the area of the 2xS group. An outlier is one diploid individual, which in all analyses grouped with the apomictic tetraploids, also placed close to them in NMDS plots. This individual had a particularly high apomictic reproductive rate (90%) in our previous flow cytometric seed screening (FCSS) study [45]. The 4xA group is clearly differentiated from the 4xM and 2x groups by a lower diversity of externally methylated but a higher diversity of internally methylated epiloci. These findings support a hypothesis of a causal relationship of methylation patterns to the mode of reproduction independent from cytotype.

AMOVAs revealed for both cytotypes and modes of reproduction a higher variation within groups than among them. Variation within groups is probably shaped not only by genotypic variability [59] but also by local micro-niches of collection sites and the age of the individual plant. However, our F_st_ values are for the methylated loci all above 0.15, which is usually regarded as the upper threshold value for epigenetic variation just within groups [68,69,70]. 

In the context of disentangling effects of ploidy from the mode of reproduction, the relative contribution of types of epiloci to the global genomic cytosine content may play a crucial role. Internal or external methylation status depends on two distinct families of methyltransferases [71,72,73], which in turn means that both pathways can be regulated independently. Furthermore, internal methylation is found more in gene bodies, while external methylation appears typically in repetitive regions and transposons [13]. In *R. kuepferi*, the internally-methylated epiloci differ between the two cytotypes with the same mode of reproduction (Table 3). Internally-methylated epiloci usually have a more heritable yet conserved methylation profile [67,74,75]. The tetraploid cytotype of *R. kuepferi* may have stabilized such profiles after multiple origins [63]. Besides the genetic and physiological background, polyploidy also seems to affect methylation patterns [76,77]. Moreover, polyploids have more sites where methylations can occur, and hence more flexibility for possible epigenetic alterations that are relevant to gene expression [78]. 

The mode of reproduction appears to relate in *R. kuepferi* additionally to external methylations, which are in general less stable and only partly heritable [67,79]. Externally methylated epiloci differentiate tetraploids with the mixed and obligate asexual mode of reproduction, mostly by a drastic loss of diversity in the latter group (Table 2, Figure 2). Although we cannot infer a functional relationship between our anonymous methylation data and expression of sexuality and apomixis, our markers suggest that obligate apomixis is connected to a specific methylome within the same ploidy level. Such a specific methylome is not apparent in the mixed apomicts, where the developmental pathway is still flexible.

Development and germline differentiation are in plants strongly controlled by epigenetic pathways [27,31,80]. High rates of sexuality among mixed reproducing individuals within either di- or tetraploids [45] may be correlated to similar methylation patterns, as these may be involved in similar genetic regulation networks necessary for the preservation of sexual pathways. Apomixis in *R. kuepferi* follows the aposporous pathway [62], which means that meiosis is completely bypassed and a somatic cell undergoes embryo sac development and that the egg cell develops without fertilization. Grimanelli [27] proposed that aposporous apomixis is likewise under epigenetic control. Hence, we hypothesize that the transition from mixed to obligate apomixis in tetraploids could be connected to alterations of methylation profiles. 

On the other hand, mode of reproduction itself can affect the inheritance of methylation patterns, as re-shuffling of potentially heritable epi-alleles may take place to some extent during sexual reproduction [74,81]. This mechanism would still operate in the category of a mixed mode of reproduction, independent from ploidy. In contrast, in obligate aposporous apomicts, meiosis and fertilization are completely bypassed and hence a certain, apomixis-specific methylation profile could be faithfully transmitted to the next generation. Such a transgenerational inheritance of a certain methylome would explain the striking divergence of all types of epiloci in obligate apomictic tetraploids from the mixed ones in *R. kuepferi*, but needs further investigations.

Strikingly, we found the described different methylation patterns related to the mode of reproduction in basal leaves (flowers could not be sampled as they were used to determine the mode of reproduction). This methylation differentiation in vegetative tissues is in line with a hypothesis by Hörandl and Hadacek [82] and experimental work [52] that the physiological status of the whole plant has an influence on the expression of apomixis. Klatt et al. [52] observed increasing proportions of apomictic seed formation in diploid *R. kuepferi* under controlled experimental cold conditions. Likewise, Reference [28] found in seedlings of obligate apomictic *Boechera* global changes of expression of both meiotic and stress response genes compared to sexual ones, whereby gene deregulation could be induced by global DNA demethylation. In the facultative apomictic grass *Eragrostis curvula*, stress treatments induced higher proportions of sexuality, and concomitant changes in cytosine methylation patterns [29]. Experimental temperature treatments in climate growth chambers do confirm a change of methylation patterns in *R. kuepferi* [25]. In this context is notable that the obligate 4x apomictic plants of *R. kuepferi*, occurring at the highest elevations [45], showed a different methylation pattern than their diploid progenitors and the mixed reproducing tetraploids in all epiloci (Figure 2). Hence, the 4xA group might represent lineages that have experienced regular cold conditions, which caused higher dynamics of methylation changes than in all other groups. These methylation changes could reflect a general response to cold stress [83]. Functional relations to the expression of apomixis, however, need to be studied on reproductive tissues and in combination with gene expression analyses.

### 3.2. Geographical Patterns and Environmental Correlations

The tetraploid cytotype of *R. kuepferi* exhibits an extended biogeographical and elevational distribution [45,58,60]. Kirchheimer et al. [60] showed that the niche optimum of tetraploids is shifted to cooler conditions at higher altitudes compared to diploids. This shift may have been driven by changes in the reproductive system of their originally warm-adapted diploid progenitors during postglacial recolonization of higher regions in the Alps. In addition, polyploidization may have increased physiological tolerance due to whole-genome duplication [84]. Alpine habitats are harsh for plant life because of low temperatures down to freezing, short growth periods, strong wind and high UV-radiation exposure [85]. Simulations of postglacial migration and niche preference suggested that the tolerance to cooler conditions, including freezing, allowed tetraploids to surmount high elevation barriers and establish new populations throughout a greater distribution range [61].

Interestingly, our methylation patterns do not reflect geographical differentiation. Values of Moran’s *I* around zero among di- and tetraploids indicate that no spatial auto-correlation exists across the entire range of the species (Table 4). The entirely diploid populations are geographically very close and have a smaller elevational range than tetraploid populations. This is in sharp contrast to population genetic structure in which the diploid sexuals showed a distinct geographical differentiation in their refugial areas with six genetic partitions, and isolation by distance [59]. Hence, we can hypothesize that the overall warmer niche in the southwestern Alps contributed to the more homogeneous epigenetic pattern. On the other hand, tetraploids likewise showed neither relevant geographic structuring nor isolation by distance in their three genetic partitions [59]. Likewise, no geographical structure appeared in our methylation patterns. Rapid postglacial recolonization of the Alps and recombination via facultative sexuality could have homogenized both genetic and epigenetic patterns. According to our results so far [45,57,63], tetraploid individuals could have emerged randomly and repeatedly from diploids by a triploid bridge in the southwestern Alps; these polyploidization events were documented previously by detailed FCSS studies [57]. Multiple origins, rapid postglacial colonization of the high elevation areas of the central and eastern Alps and regular intermixing due to facultative sexuality among tetraploids could have facilitated a geographical uniformity of methylation patterns. 

In contrast, we found high values of Moran’s *I* (>0.5) in correlation to altitude, as well as to annual mean temperature among di- and tetraploid populations (Table 5). We found greater variation in terms of temperature, but a larger overall impact of altitude on methylation patterns. Most of the correlations to these ecological variables were found among the less stable externally methylated epiloci. The diploid populations in south-western France exhibit a smaller altitudinal range compared to the tetraploid populations. The correlation of Moran’s *I* with annual precipitation is slightly lower because the Alps in south-west France have a relatively warm and dry climate. Thus, calculated correlations suggest the effects of climatic conditions, especially of cold temperature regimes at high elevations, on methylation patterns. Effects of cold stress and cold acclimation on methylation patterns in plants have been demonstrated in many experimental studies in plants [83], and also in *R. kuepferi* [25]. 

**Figure 3:**
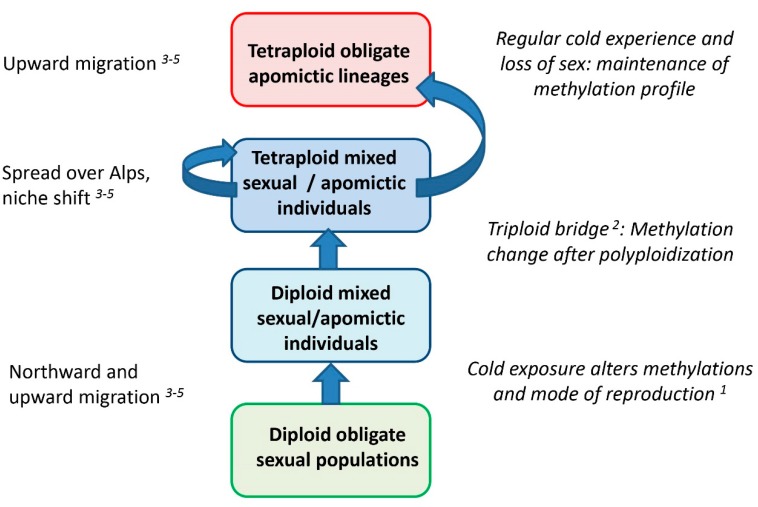
Hypothetical evolutionary scenario of the interplay of climatic conditions, polyploidization, and shifts to apomixis with methylation patterns, integrating previous publications: *^1^* [52], *^2^* [57], *^3^* [45], *^4^* [60], *^5^* [61].

The results support our hypotheses that changes of methylation patterns correlate in *R. kuepferi* not only to polyploidization and to shifts to apomixis, but also to climatic conditions. By integrating previous information from five publications we present the following hypothetical scenario (Figure 3): warm-adapted diploid *R. kuepferi* in the southwestern Alps experienced colder conditions during postglacial northward migration, thereby shifting to mixed reproduction [52]. The subsequent polyploidization [57] resulted in an altered methylation profile, characterizing mostly the mixed tetraploids. These processes happened probably in the sympatric zone of diploids and tetraploids [45,57]. Colonization of the higher elevations of the Alps by tetraploids und the niche shift to colder climates [60,61] resulted in obligate apomixis [45,52] with a concomitant change of the methylation profile. Hence, we tentatively conclude that epigenetic patterns reflect a stepwise change according to an ecological gradient rather than a geographical differentiation. 

## 4. Materials and Methods

### 4.1. Plant Material

Plants of *Ranunculus kuepferi* were collected from 81 localities throughout the whole distribution range in the Alps during field trips in 2013/14 and transferred to the Botanical Garden of the University of Goettingen as previously described [45,57]. Herbarium specimens have been deposited in the collections of the Herbarium of the University of Goettingen (GOET). Leaves of 1074 individuals were collected directly in the field and dried in silica gel, to preserve methylation patterns of the conditions of the natural sites, and to prevent the influence of digging out and transfer of plants. Ploidy levels of individuals were identified via flow cytometry with silica gel dried leaf material, while reproduction modes from seeds collected in the wild were determined using flow cytometric seed screening (FCSS; [86,87]) on five seeds per individual from 551 plants which provided enough seed material [45]. Methods of FCSS are given in Supplementary Methods S1 and results in Supplementary Table S4. Since we wanted to study methylation patterns under natural conditions over a large distribution area in the Alps, we relied on a comprehensive sampling of silica gel-dried leaf material collected in the field; reproductive structures could not be sampled for epigenetic analysis because flowers/fruiting heads were needed for FCSS analysis (see above). *Ranunculus kuepferi* has a relatively big genome size (1C = 4.4 pg DNA; [58]), and no reference genome is available. Many of the silica gel-dried samples did not provide sufficient quality and quantity of DNA extracts for bisulfite sequencing protocols. Hence, we preferred methylation-sensitive AFLPs (MSAP) as a well-established, robust method for getting an overview of a representative set of samples for non-model organisms without a reference genome [13,67] over a more functionally orientated bisulfite-based sequencing approach [22,88]. We categorized individuals according to cytotype (2x, 4x) and reproduction mode (obligate sexual, mixed, obligate apomicts) according to [45]. “Obligate” means that a plant produced exclusively sexual (S) or apomictic seeds (A), while “mixed” is defined that a plant produced both sexual and apomictic seeds (see Reference [45] for developmental pathways and terminology). We further defined four combined groups: obligate sexual diploids (2xS), facultative apomictic diploids (2xM), obligate apomictic tetraploids (4xA) and facultative sexual tetraploids (4xM). From these 551 plants, we chose 48 individuals of each group for MSAP analysis except for the 2xM group, as only six diploid individuals exhibited apomictic seed production. The sampling aimed at covering the whole distribution area but was random with respect to the mode of reproduction. From these 150 individuals, 27 (18%; 7 diploids, 20 tetraploids) were excluded from further steps because of the low quality of MSAP electropherograms, resulting in a final dataset of 41 diploid, sexual (2xS), 6 diploid, mixed (2xM), 45 tetraploid, mixed (4xM) and 31 tetraploid, apomictic (4xA) scored samples. The samples belong to 48 populations out of the whole Alps, with 1–6 individuals per population. A list of the 123 individuals used for MSAP analyses with population/sample ID, provenances, and ploidy level/mode of reproduction is given in Supplementary Table S4. 

### 4.2. Methylation-Sensitive Amplified Fragment-Length Polymorphisms

We extracted DNA with the QIAGEN DNeasy Plant Mini Kit (QIAGEN, Hilden, Germany) using a slightly modified protocol (see details in Supplementary Methods S2). Detection of epigenetic patterns was accomplished by conducting methylation-sensitive amplified fragment-length polymorphisms (MSAP). We followed the protocol of [18] with some minor modifications (see below).

We performed digestion and ligation of DNA subsequently as two independent reactions on each sample using different restriction enzyme combinations: (i) HpaII (New England Biolabs, Ipswich, MA, USA) as a frequent cutter and EcoRI (New England Biolabs, Ipswich, MA, USA) as a rare cutter, (ii) MspI (New England Biolabs, Ipswich, MA, USA) and EcoRI. Both combinations (containing per sample: 3.38 µL ddH_2_O, 1.15 µL NEB CutSmart Buffer, 1.15 µL NEB MspI or HpaII respectively, 0.07 µL NEB EcoRI-HF) were run in parallel under same conditions each with 5.75 µL of the same DNA isolate. HpaII and MspI are isoschizomeres with the same recognition sequence (5′-CCGG-3′). Both enzymes cut a nonmethylated restriction site, and MspI cuts also if only the internal cytosine is either holo- or hemimethylated [89]. Cleaving of HpaII is entirely blocked if either one or both cytosines are holomethylated, whereas hemimethylation on either or both cytosines only impairs restriction [89], which can be overcome with high fidelity enzymes, optimal digestion conditions and a prolonged incubation time. Digestion was performed at 37 °C in a T100 Thermocycler (Bio-Rad Laboratories Inc., Hercules, CA, USA) for 1 h.

Following digestion, adapters (Eurofins Scientific, Brussels, Belgium; see Supplementary Table S5) were ligated adding 11.5 µL reaction mix (containing per sample: 3.1 µL ddH_2_O, 2.3 µL NEB T4 Ligase Buffer, 2.8 µL 0.5M NaCl, 1.15 µL NEB MspI or HpaII respectively, each 0.9 µL EcoRI and MspI/HpaII adapter mix, 0.35 µL NEB T4 Ligase) and incubated for another hour at 37 °C and subsequently overnight at 16 °C. Products were analyzed on a 1.5% agarose gel and diluted 10-fold prior to preselective amplification. 

The first step reduction of fragments with preselective primers (Eurofins Scientific, Brussels, Belgium; see Supplementary Table S5) was performed with a slightly modified master mix (containing per sample: 6.3 µL ddH_2_O, 1.5 µL NEB OneTaq Buffer, 1.5 µL MgCl_2_, 0.3 µL dNTP mix 25µM each, 0.7µL primer mix, 0.2 µL NEB OneTaq) adding 4.5 µL of diluted R/L product on a T100 Thermocycler (120s 94 °C; 20x 10s 94 °C, 30s 62 °C, 120s 72 °C; 30 min 60 °C; ∞ 4 °C). Products were checked on a 1.5% agarose gel and diluted 10-fold prior to selective amplification. 

We used three selective HpaII/EcoRI primer combinations, each with three selective nucleotides for HpaII and two for EcoRI, adapted from previous AFLP studies on *R. kuepferi* [59]: HpaII+TCG/EcoRI+AC, HpaII+TGA/EcoRI+AC, HpaII+ATA/EcoRI+AC (Eurofins Scientific, Brussels, Belgium; see Supporting information Table S5). Three separate PCRs were conducted in parallel with the same master mix (containing per sample: 6.6 µL ddH_2_O, 1.5 µL NEB OneTaq Buffer, 1.5 µL MgCl_2_, 1.0 µL dNTP mix 25 µM each, 0.6 µL EcoRI primer, 0.6µL selective primer, 0.2µL NEB OneTaq) adding 3 µL of preselective amplification product on T100 Thermocyclers (120s 94 °C; 9x 10s 94 °C, 30s 67 °C–1 °C per cycle, 120s 72 °C; 23x 10s 94 °C, 30s 62 °C, 120s 72 °C; 30 min 60 °C; ∞ 4 °C). Products were kept cool at 4 °C overnight and diluted 20-fold prior to fragment analysis.

Fragment analyses were performed on an ABI Prism 3730 capillary sequencer (Applied Biosystems, Waltham, MA, USA) using GeneScan ROX 500 (Thermo Fisher Scientific, Waltham, MA, USA) as an internal size standard. Fragment quality was first checked visually with GeneMarker 2.7.4 software (SoftGenetics LLC., State College, PA, USA). As AFLP methods are prone to false positive fragment peaks, and also because of potential bias of silica-gel dried materials, we produced duplicates of every sample from restriction to selective PCR, to ensure 100% reproducibility of the resulting electropherograms (see also subsection “statistical data evaluation”).

Analysis and interpretation of methylation patterns among samples and groups are based on present/absent profiles of fragments. To overcome the subjectivity of manual scoring [90], transformation of fragments between 100 and 600 bp to dominant binary matrices was conducted automatically using Peakscanner 2.0 software (Applied Biosystems, Waltham, MA, USA) for basic peak detection. The R package RawGeno 2.0-2 (Available online: http://sourceforge.net/projects/rawgeno) [90] was used for fragment identification as well as filtering of technical artifacts and non-reproducible fragments. To find optimal parameter combinations we ran a script implemented in RawGeno incrementally increasing stepwise every parameter and calculating relevant actuating factors on analysis quality (final parameter settings, reproducibility and error rates are given in Supplementary Table S6). Raw fragment recognition data from RawGeno were imported into the MSAP_calc.r script that distinguishes HpaII and MspI profiles, and filters for susceptible loci as described in Reference [89]. Based on the differing sensitivity of the restriction enzyme isochizomers to methylation of their target sequence, four conditions can be distinguished: (i) no methylation (both HpaII and MspI cut), (ii) holo- or hemimethylation of internal cytosine (C_Me_CGG or C_HMe_CGG, respectively, only MspI cuts), (iii) hemimethylation of external cytosine (_HMe_CCGG, only HpaII cuts), iv) either holomethylation of both internal and external cytosine or a mutation (both HpaII and MspI do not cut). To transform fragment patterns into a 0/1 matrix for further analyses, we chose the Mixed Scoring 2 approach [89]. For this purpose, each locus was divided into three epiloci (nonmethylated, internally C_Me_CGG/C_HMe_CGG-methylated, externally _HMe_CCGG-methylated): (i) nonmethylated was scored as “100” (ii) internally-methylated as “010”, and (iii) externally-methylated as “001”. Condition (iv) was scored as “000”, as it represents an ambiguous methylation or mutation status, which is not distinguishable [89]. Only conditions (i) to (iii) were used for further statistical analysis. The restriction enzyme isochizomer reactions and their respective scoring were analyzed independently, and only afterward data were combined. One cannot infer changes from one type of epiloci to the other [91], but for reporting patterns of methylations in non-model organisms this method is well established [13].

### 4.3. Statistical Data Evaluation

Statistical analyses were performed in R version 3.4.2 (Available online: https://cran.r-project.org/bin/windows/base/old/3.4.2/) (R Foundation for Statistical Computing, Vienna, Austria) on the basis of the presence/absence matrix (Supplementary Table S7) for 1088 epiloci (see above). We tested separately for the factors ploidy (2x/4x) and mode of reproduction (sexual, mixed, apomictic) by calculating AMOVAs and one-factorial ANOVAs on the presence/absence matrix. For combined groups, descriptive parameters were adopted from RawGeno in R 2.15.3 and further explored in Rcmdr 2.4-4 (Available online: http://socserv.socsci.mcmaster.ca/jfox/Misc/Rcmdr/) [92]. Percentages were arcsine transformed to match a normal distribution of data. Pairwise ANOVAs between combined groups were carried out in R using descriptive parameters of polymorphic loci distribution and abundance.

We used non-metric multidimensional scaling with non-Euclidean Jaccard distances in vegan 2.4-5 (Available online: https://github.com/vegandevs/vegan) [93] and ggplot2 3.2.1 (Available online: https://github.com/tidyverse/ggplot2) [94] to visualize grouping of individuals according to their methylation patterns. We calculated nine AMOVAs to compare the molecular epigenetic variances within and among our predefined combined groups (2xS, 2xM, 4xM, 4xA), as well as the ploidy levels (diploid, tetraploid) and different reproduction modes (sexual, mixed, apomictic). For F_st_ values, as measures of genetic divergence), based on the three different types of methylation (non-, internally-, externally methylated see Supplementary Table S4). In addition, we determined the epigenetic phenotypic differentiation (ΦST) of loci by means of locus-by-locus AMOVA analyses. All AMOVAs were executed in ARLEQUIN 3.5.22 (Swiss Institute of Bioinformatics, Bern, Switzerland) [95]. We have calculated each for haplotypic data, a gamma of 0.0 and 50,000 permutations.

We tested for potential correlations between individual methylation patterns and spatial distribution with a stratified Mantel test in R with ecodist 2.0.1 (Available online: https://github.com/phiala/ecodist) [96] using mismatch coefficients as suitable dissimilarity distances for dominant marker data. We calculated geographic distances from population GPS centroid data. We furthermore calculated Moran’s *I* [97] to examine global spatial structuring over the entire sampling area, as well as Geary’s *C* [98] for more detailed local structure analysis. Moran’s *I* values range between −1 and +1, whereby positive values indicate global spatial autocorrelation, a value near 0 indicates random distribution, and negative value perfect dispersal. Geary’s *C* values explain local spatial autocorrelation, the values are always positive (>0; Supplementary Table S5). The main environmental parameters for the distribution of the cytotypes (elevation, annual mean temperature, precipitation) were selected according to the study of [60] and data were downloaded from the WorldClim 1.4 database (Available online: https://worldclim.org/data/v1.4/worldclim14.html) [99]. The correlation of these parameters with observed methylation patterns was investigated with Samßada 0.5.3 (Available online: http://lasig.epfl.ch/sambada) [100]. Samßada uses an approach similar to logistic regressions to model the probability of observing a particular genotype of a polymorphic marker given the environmental conditions at the sampling locations [100] (at the 100 × 100 m scale of [60]), returning local Moran’s *I* values as output. Our chosen multivariate approach with three environmental predictor variables was similar to a forward stepwise regression. Furthermore, we tested for associations between putative candidate epiloci and environmental parameters using a logistic regression for univariate models, with model selection based on Wald and G test statistics as implemented in Samßada. The resulting β-parameters (one constant parameter corresponding to the marker, and one corresponding to the environmental variable) were used for regressions (Supplementary Table S6 and Figures S1 and S2).

## Figures and Tables

**Table 1:** Hierarchical AMOVA (analysis of molecular variance) of epiloci with cytotype as first and reproduction mode as the second level.

	SSD	MSD	df	Variance Coefficients	Sigma^2^	*p*
Cytotype	7.437	7.437	1	45.46	0.059	<0.001
Reproduction mode	6.036	6.036	2	34.92	0.127	<0.001
Error	31.135	0.259	120	46.37	0.259	
Total	44.609	0.366	122			

SSD: Squared standard deviation, MSD: Mean standard deviation, df: Degrees of freedom, Sigma^2^: representation of variance measures, *p*: *p*-value.

**Table 2:** Descriptive statistical parameters of variation of epiloci of combined groups.

	Nonmethylated						
	N_Marker_						H’_Shannon_
	Total	Polym	[%]_Poly_	Private	[%]_Priv_		Total
2xS (41)	268	162	60.5	40	24.7		0.285
2xM (6)	268	85	31.7	6	7.1		0.248
4xM (45)	268	190	70.9	64	33.7		0.326
4xA (31)	268	48	17.9	2	4.2		0.109
	**externally methylated**						
	N_Marker_						H’_Shannon_
	Total	Polym	[%]_Poly_	Private	[%]_Priv_		Total
2xS (41)	378	260	68.78	53	20.38		0.323
2xM (6)	378	180	47.62	14	7.78		0.358
4xM (45)	378	242	64.02	56	23.14		0.311
4xA (31)	378	62	16.40	6	9.68		0.088
	**internally methylated**						
	N_Marker_						H’_Shannon_
	Total	Polym	[%]_Poly_	Private	[%]_Priv_		Total
2xS (41)	442	260	58.82	46	17.69		0.235
2xM (6)	442	139	31.45	4	2.88		0.228
4xM (45)	442	197	44.57	20	10.15		0.173
4xA (31)	442	309	69.91	69	22.33		0.325

N_Marker_: number of markers, (Polym: total number of polymorphic markers, [%]_Poly_: percentage of polymorphic markers, Private: number of private polymorphic markers, [%]_Priv_: percentage of private polymorphic markers), H’_Shannon_: Mean Shannon diversity index. 2xS: diploid obligate sexual, 2xM: diploid mixed; 4xM: tetraploid mixed; 4xA: tetraploid obligate apomictic. The number of individuals per group is given in brackets.

**Table 3:** Pairwise comparisons of combined groups with ANOVA (analysis of variance) for the different types of epiloci in *R. kuepferi*.

	Pair			EST	SE	*t*	*p*	Adjusted *p*
**Nonmethylated**	***2xS***	***-***	***2xM***	**10.365**	**2.967**	**3.494**	**0.004**	**0.004**
	*2xS*	*-*	*4xM*	−6.311	1.473	−4.284	<0.001	<0.0012
	*2xS*	*-*	4xA*	22.439	1.615	13.891	<0.001	<0.0012
	***2xM***	***-***	***4xM***	**−16.676**	**2.954**	**−5.646**	**<0.001**	**<0.0012**
	*2xM*	*-*	4xA*	12.074	3.027	3.989	<0.001	<0.0012
	***4xM***	***-***	4xA*	**28.75**	**1.591**	**18.065**	**<0.001**	**<0.0012**
**Externally Methylated**	***2xS***	***-***	***2xM***	**−4.935**	**3.736**	**−3.736**	**0.536**	**0.6432**
	*2xS*	*-*	*4xM*	−3.423	1.855	−1.855	0.245	0.3675
	*2xS*	*-*	4xA*	−50.212	2.035	−2.035	<0.001	<0.002
	***2xM***	***-***	***4xM***	**1.513**	**3.72**	**−3.72**	**0.976**	**0.976**
	*2xM*	*-*	4xA*	−45.277	3.812	−3.812	<0.001	<0.002
	***4xM***	***-***	4xA*	**−46.79**	**2.004**	**−2.004**	**<0.001**	**<0.002**
**Internally Methylated**	***2xS***	***-***	***2xM***	**−5.43**	**3.508**	**−1.548**	**0.397**	**0.397**
	*2xS*	*-*	*4xM*	9.734	1.742	5.588	<0.001	<0.0015
	*2xS*	*-*	4xA*	27.772	1.91	14.541	<0.001	<0.0015
	***2xM***	***-***	***4xM***	**15.164**	**3.492**	**4.342**	**0.002**	**0.0024**
	*2xM*	*-*	4xA*	33.202	3.579	9.277	<0.001	<0.0015
	***4xM***	***-***	4xA*	**18.038**	**1.882**	**9.586**	**<0.001**	**<0.0015**

Pairs of groups with either equal ploidy or equal mode of reproduction are marked in bold, pairs with significant differences in all epiloci are marked with an asterisk. EST: Estimate, SE: Standard Error, *t*: t-value, *p*: *p*-value; adjusted *p*-value after [64]; 2xS: diploid obligate sexual, 2xM: diploid mixed; 4xM: tetraploid mixed; 4xA: tetraploid obligate apomictic. - denotes the pairs (e.g. 4xM and 4xA).

**Table 4:** Moran’s *I* values of epiloci in correlation to global spatial structure in combined groups of *R. kuepferi*.

	2xS					2xM				4xM				4xA		
	*I_obs_*	*I_est_*	*p*	Adjusted *p*	*I_obs_*	*I_est_*	*p*	Adjusted *p*	*I_obs_*	*I_est_*	*p*	Adjusted *p*	*I_obs_*	*I_est_*	*p*	*Adjusted p*
nonmethylated	0.201	−0.008	0.462	0.472	0.028	−0.004	0.468	0.482	0.042	−0.008	0.439	0.451	0.058	−0.009	0.441	0.444
externally methylated	0.191	−0.008	0.455	0.472	0.047	−0.009	0.475	0.482	0.062	−0.007	0.446	0.451	0.044	−0.008	0.444	0.444
internally methylated	0.136	−0.007	0.472	0.472	0.027	−0.007	0.482	0.482	0.013	−0.008	0.451	0.451	0.062	−0.008	0.444	0.444

(2xS: Diploid, sexual; 2xM: Diploid, mixed; 4xM: Tetraploid, mixed; 4xA: Tetraploid, apomictic) and methylation status comparisons. I_obs_ = Observed Moran’s *I*, I_est_ = Estimated Moran’s *I*. *p* = *p* value, adjusted *p* value after [64].

**Table 5:** Moran’s *I* values of epiloci for cytotypes in correlation to observed environmental parameters.

	Altitude	AMT	AP
Diploids	0.845	0.789	0.751
Tetraploids	0.700	0.682	0.791

AMT: Annual mean temperature (BIO1, WorldClim), AP: Annual precipitation (BIO12, WorldClim).

## Supplementary Materials

Supplementary materials can be found at https://www.mdpi.com/1422-0067/21/9/3318/s1.

## Abbreviations

MSAP	methylation sensitive amplified fragment-length polymorphisms
