# Supplementary material for: Epigenetic Patterns and Geographical Parthenogenesis in the Alpine Plant Species Ranunculus kuepferi (Ranunculaceae)
Source: Int J Mol Sci. 2020 May 7;21(9):3318. doi: 10.3390/ijms21093318 (PMC7247541; doi:10.3390/ijms21093318)
Supplement: Supplementary file 1 [file ijms-21-03318-s001.pdf]

Article

Epigenetic Patterns and Geographical Parthenogenesis in the Alpine Plant Species *Ranunculus kuepferi* (Ranunculaceae)

Christoph C. F. Schinkel, Eleni Syngelaki, Bernhard Kirchheimer, Stefan Dullinger, Simone Klatt and Elvira Hörandl

The following supplementary material is available for this article:

Table S1. Results of molecular variance analyses (AMOVA) of epiloci. df: Degrees of freedom, p: p-value.

		Non-methylated			
Source of variation	df	Variance components	*p*	% variation	*F* statistics
Among combined groups	3	11.510	<0.001	42.95	F_{ST}: 0.526
Within groups	119	15.287	<0.001	57.05	
Among cytotypes	1	7.375	<0.001	27.11	F_{ST}: 0.533
Within cytotypes	121	19.828	<0.001	72.89	
Among reproduction modes	2	7.129	<0.001	26.14	F_{ST}: 0.534
Within reproduction modes	120	20.149	<0.001	73.86	
		Internally-methylated			
Source of variation	df	Variance components	*p*	% variation	*F* statistics
Among groups	3	10.900	<0.001	32.98	F_{ST}: 0.448
Within groups	119	22.151	<0.001	67.02	
Among cytotypes	1	11.501	<0.001	32.15	F_{ST}: 0.491
Within cytotypes	121	24.275	<0.001	67.85	
Among reproduction modes	2	10.515	<0.001	29.57	F_{ST}: 0.487
Within reproduction modes	120	25.047	<0.001	70.43	
		Externally-methylated			
Source of variation	df	Variance components	*p*	% variation	*F* statistics
Among groups	3	8.526	<0.001	27.68	F_{ST}: 0.366
Within groups	119	22.278	<0.001	72.32	
Among cytotypes	1	5.011	<0.001	16.23	F_{ST}: 0.367
Within cytotypes	121	25.859	<0.001	83.77	
Among reproduction modes	2	4.531	<0.001	14.74	F_{ST}: 0.365
Within reproduction modes	120	26.216	<0.001	85.26	

Table S2. Geary's C values correlated to environmental variables.

Population	Group	Altitude	Mean Annual Temp.	Annual Precipitation
3	2xS	2.550	2.577	2.232
23	2xS	0.676	1.869	2.464
24	2xS	0.081	0.055	0.021
25	2xS	2.164	0.366	0.001
26	2xS	1.948	0.279	0.021
27	2xS	2.019	0.464	0.010
29	2xS	0.391	0.378	0.072
33	2xS	2.992	3.090	2.613
31	2xS	0.108	0.695	0.696
233	2xS	1.481	1.499	1.008
235	2xS	0.092	0.055	0.021
202	2xS	0.003	0.090	0.218
203	2xS	0.014	0.221	0.371
204	2xS	0.278	0.596	0.768
206	2xS	0.532	1.149	1.008
201	2xS	0.414	0.378	0.153
207	2xS	0.073	0.214	0.153
112	2xS	0.618	1.475	2.094
115	2xS	0.027	0.000	0.010
117	2xS	0.585	1.666	2.464
26	2xM	1.948	0.279	0.021
31	2xM	0.108	0.695	0.696
204	2xM	0.278	0.596	0.768
207	2xM	0.073	0.214	0.153
201	4xM	0.027	0.108	3.264
36	4xM	0.158	0.404	0.288
37	4xM	0.332	0.336	0.342
40	4xM	3.285	1.067	0.609
48	4xM	0.018	0.108	0.010
54	4xM	0.112	0.108	2.012
58	4xM	0.321	0.057	1.876
75	4xM	4.632	3.252	0.860
17	4xM	0.356	0.016	0.535
17	4xM	0.356	0.016	0.535
79	4xM	0.050	2.140	0.116
83	4xM	0.032	0.612	0.060
81	4xM	2.004	2.140	0.152
204	4xM	8.287	7.480	1.618
205	4xM	0.023	0.562	0.022
114	4xM	0.259	0.124	0.059
20	4xA	0.027	0.108	3.264
36	4xA	0.158	0.404	0.288
48	4xA	0.018	0.108	0.010
54	4xA	0.112	0.108	2.012
75	4xA	4.632	3.252	0.860
17	4xA	0.356	0.016	0.535
79	4xA	0.050	2.140	0.116
83	4xA	0.032	0.612	0.060

205	4xA	0.023	0.562	0.022
104	4xA	0.096	0.148	1.056
114	4xA	0.259	0.124	0.059
111	4xA	0.002	0.336	0.085
81	4xA	2.004	2.140	0.152

Table S3. Candidate loci and environmental correlations as revealed by Samßada. NON = nonmethylated.M = internally methylated.EXT = externally methylated.Env = environmental variable: ALT = elevation, MEAN = Mean annual temperature. Error code (0 if success). AIC = Akaike information criterion, BIC = Bayesian information criterion; Beta_0 = constant parameter (corresponding to marker), Beta_1 = parameter corresponding to environmental variable.

Marker	Env	Loglikelihood	Gscore	WaldScore	NumError	AIC	BIC	Beta_0	Beta_1
EXT154	ALT	-57.493	45.430	29.094	0	118.986	134.235	9.932	-0.005
EXT17	ALT	-55.971	41.720	28.377	0	115.942	131.191	9.077	-0.005
EXT197	ALT	-52.083	36.847	26.700	0	108.167	123.415	8.171	-0.005
EXT199	ALT	-65.634	35.643	24.769	0	135.268	150.517	8.588	-0.004
EXT210	ALT	-45.748	35.387	25.682	0	95.495	110.744	8.067	-0.005
EXT235	ALT	-65.402	35.384	24.791	0	134.804	150.053	8.479	-0.004
EXT241	ALT	-55.319	36.274	26.438	0	114.638	129.887	8.080	-0.004
EXT248	ALT	-55.858	31.345	24.159	0	115.717	130.965	7.246	-0.004
EXT33	ALT	-50.375	55.833	31.731	0	104.749	119.998	11.722	-0.006
EXT351	ALT	-54.694	56.009	30.682	0	113.389	128.637	12.356	-0.006
EXT358	ALT	-59.037	34.016	25.298	0	122.074	137.323	7.749	-0.004
EXT52	ALT	-66.965	34.750	24.005	0	137.930	153.179	8.630	-0.004
EXT74	ALT	-55.339	41.413	28.331	0	114.677	129.926	8.998	-0.005
EXT86	ALT	-55.145	51.261	30.437	0	114.290	129.538	11.075	-0.006
EXT95	ALT	-53.405	49.772	30.557	0	110.811	126.059	10.575	-0.006
M92	ALT	-67.984	34.538	22.872	0	139.968	155.216	9.183	0.004
NON129	ALT	-65.708	37.264	24.969	0	135.416	150.665	9.102	-0.004
NON168	ALT	-55.070	50.276	30.350	0	114.140	129.389	10.825	-0.006
NON173	ALT	-57.027	41.105	28.087	0	118.055	133.304	9.001	-0.005
NON180	ALT	-62.894	33.423	24.666	0	129.787	145.036	7.796	-0.004
NON250	ALT	-46.971	32.941	24.647	0	97.942	113.190	7.637	-0.005
NON47	ALT	-57.493	38.676	27.245	0	118.986	134.235	8.557	-0.005
EXT137	MEAN	-49.271	33.416	23.118	0	102.541	117.790	-3.160	0.064
EXT154	MEAN	-44.554	71.307	35.385	0	93.109	108.358	-3.272	0.099

EXT159	MEAN	-48.665	37.026	24.752	0	101.329	116.578	-3.236	0.067
EXT17	MEAN	-46.162	61.338	33.658	0	96.324	111.573	-3.327	0.089
EXT186	MEAN	-52.844	43.028	28.485	0	109.688	124.936	-2.845	0.069
EXT197	MEAN	-46.092	48.829	29.175	0	96.185	111.434	-3.492	0.080
EXT199	MEAN	-58.851	49.209	31.336	0	121.702	136.951	-2.041	0.069
EXT210	MEAN	-42.494	41.895	24.998	0	88.988	104.237	-3.883	0.079
EXT216	MEAN	-53.510	39.891	27.152	0	111.021	126.269	-2.803	0.066
EXT227	MEAN	-49.330	35.694	24.262	0	102.661	117.910	-3.170	0.066
EXT235	MEAN	-56.363	53.462	32.581	0	116.726	131.974	-2.221	0.074
EXT24	MEAN	-48.821	34.316	23.471	0	101.641	116.890	-3.205	0.065
EXT241	MEAN	-47.142	52.627	31.015	0	98.285	113.534	-3.346	0.082
EXT248	MEAN	-49.344	44.373	28.291	0	102.689	117.938	-3.175	0.073
EXT33	MEAN	-42.905	70.772	34.984	0	89.811	105.060	-3.539	0.100
EXT351	MEAN	-40.373	84.652	34.728	0	84.745	99.994	-3.392	0.117
EXT358	MEAN	-52.835	46.420	29.958	0	109.670	124.919	-2.813	0.071
EXT52	MEAN	-62.764	43.152	29.102	0	129.528	144.777	-1.720	0.063
EXT56	MEAN	-66.457	33.275	24.867	0	136.913	152.162	-1.657	0.053
EXT74	MEAN	-45.653	60.783	33.338	0	95.307	110.555	-3.403	0.089
EXT76	MEAN	-52.246	40.537	27.218	0	108.491	123.740	-2.914	0.067
EXT86	MEAN	-43.512	74.527	35.525	0	91.025	106.273	-3.315	0.103
EXT95	MEAN	-44.856	66.870	34.715	0	93.713	108.962	-3.368	0.095
M228	MEAN	-60.558	40.436	25.154	0	125.116	140.364	0.618	-0.065
M290	MEAN	-64.360	34.891	23.684	0	132.721	147.970	0.594	-0.057
M32	MEAN	-56.806	55.526	30.159	0	117.612	132.861	1.359	-0.080
M92	MEAN	-60.708	49.091	30.271	0	125.415	140.664	1.530	-0.069
NON129	MEAN	-56.764	55.152	32.641	0	117.529	132.778	-2.039	0.076
NON131	MEAN	-56.839	47.873	31.013	0	117.678	132.927	-2.360	0.069
NON157	MEAN	-51.815	33.033	23.407	0	107.629	122.878	-2.945	0.061
NON168	MEAN	-44.356	71.704	35.403	0	92.712	107.961	-3.289	0.099
NON171	MEAN	-64.642	32.267	24.342	0	133.284	148.533	-1.883	0.052

NON173	MEAN	-47.307	60.545	33.732	0	98.614	113.863	-3.198	0.087
NON180	MEAN	-54.399	50.412	31.727	0	112.798	128.046	-2.578	0.073
NON250	MEAN	-44.612	37.659	23.936	0	93.223	108.472	-3.625	0.072
NON47	MEAN	-49.710	54.242	32.306	0	103.420	118.669	-3.033	0.080

Table S4. List of the 123 individuals used for MSAP analyses with individual code (population number_plotnumber_individual number) provenances, ploidy level, respective reproductive group, and percentage of sexual seeds with n = 5-10 (data from Schinkel et al. 2016). 2x = diploid, 4x = tetraploid, 2xM = diploid mixed, 2xS = diploid obligate sexual, 4xA = tetraploid obligate apomictic, 4xM = tetraploid mixed.

	**Geographic Coordinates (WGS84)**					
**Individual**	**North**	**East**	**Elevation [m.a.s.l.]**	**Cytotype**	**Reproduction mode**	**% sexual seeds**
14_2_2	4.435.489	6.505.583	1880	2x	2xM	80
26_2_1	4.490.033	5.469	1456	2x	2xM	80
31_3_2	4.419.522	7.659.222	1937	2x	2xM	40
32_2_3	4.420.667	71.475	2320	2x	2xM	90
204_1_1	4.415.725	6.729.195	1640	2x	2xM	40
207_3_1	4.430.108	6.567.389	1921	2x	2xM	20
3_3_1	4.420.028	715.639	2291	2x	2xS	100
3_3_2	4.420.028	715.639	2291	2x	2xS	100
23_2_1	4.374.931	6.657.444	1616	2x	2xS	100
23_2_2	4.374.931	6.657.444	1616	2x	2xS	100
24_3_1	4.415.083	6.543.611	1925	2x	2xS	100
24_4_2	4.415.083	6.543.611	1925	2x	2xS	100
25_2_1	4.490.128	5.476.167	1435	2x	2xS	100
25_3_3	4.490.128	5.476.167	1435	2x	2xS	100
25_4_3	4.490.128	5.476.167	1435	2x	2xS	100
26_3_2	4.490.033	5.469	1456	2x	2xS	100
27_1_1	4.483.933	5.424.222	1449	2x	2xS	100

27_2_1	4.483.933	5.424.222	1449	2x	2xS	100
27_2_3	4.483.933	5.424.222	1449	2x	2xS	100
29_1_1	4.423.111	7.617.222	2020	2x	2xS	100
29_2_3	4.423.111	7.617.222	2020	2x	2xS	100
31_2_3	4.419.522	7.659.222	1937	2x	2xS	100
33_3_1	4.421.305	7.146.667	2328	2x	2xS	100
33_3_3	4.421.305	7.146.667	2328	2x	2xS	100
112_1_2	438.525	6.352.778	1626	2x	2xS	100
112_2_3	438.525	6.352.778	1626	2x	2xS	100
112_4_1	438.525	6.352.778	1626	2x	2xS	100
114_4_3	4.472.089	6.919.861	2339	2x	2xS	100
115_3_2	44.245	6.756.111	1891	2x	2xS	100
115_3_3	44.245	6.756.111	1891	2x	2xS	100
117_2_2	4.374.556	6.655.833	1632	2x	2xS	100
117_3_3	4.374.556	6.655.833	1632	2x	2xS	100
118_3_3	4.423.161	7.628.333	1636	2x	2xS	100
201_2_2	4.408.044	66.375	2025	2x	2xS	100
201_2_3	4.408.044	66.375	2025	2x	2xS	100
202_1_1	44.159	6.714.611	1829	2x	2xS	100
202_4_2	44.159	6.714.611	1829	2x	2xS	100
203_1_3	4.416.294	6.710.805	1840	2x	2xS	100
203_3_1	4.415.725	6.729.195	1840	2x	2xS	100
204_4_2	4.415.725	6.729.195	1640	2x	2xS	100
206_1_2	442.468	66.988	2049	2x	2xS	100
206_3_1	442.468	66.988	2049	2x	2xS	100
207_1_3	4.430.108	6.567.389	1921	2x	2xS	100
233_1_1	4.412.946	696.878	2185	2x	2xS	100
233_1_2	4.412.946	696.878	2185	2x	2xS	100
235_1_1	4.416.689	670.763	1930	2x	2xS	100
235_1_3	4.416.689	670.763	1930	2x	2xS	100

17_1_2	4.505.069	6.390.778	2357	4x	4xA	0
17_3_1	4.505.069	6.390.778	2357	4x	4xA	0
17_4_3	4.505.069	6.390.778	2357	4x	4xA	0
20_1_2	4.634.719	7.724.111	2200	4x	4xA	0
20_2_1	4.634.719	7.724.111	2200	4x	4xA	0
20_4_2	4.634.719	7.724.111	2200	4x	4xA	0
36_1_3	4.538.542	7.043.722	2152	4x	4xA	0
36_3_2	4.538.542	7.043.722	2152	4x	4xA	0
48_1_1	4.647.153	9.728.889	2262	4x	4xA	0
48_1_3	4.647.153	9.728.889	2262	4x	4xA	0
48_4_1	4.647.153	9.728.889	2262	4x	4xA	0
54_2_2	4.627.239	1.057.506	2303	4x	4xA	0
54_3_1	4.627.239	1.057.506	2303	4x	4xA	0
54_3_2	4.627.239	1.057.506	2303	4x	4xA	0
54_4_3	4.627.239	1.057.506	2303	4x	4xA	0
75_1_2	4.652.845	9.811.194	2678	4x	4xA	0
79_1_2	4.698.753	1.035.919	2280	4x	4xA	0
79_2_2	4.698.753	1.035.919	2280	4x	4xA	0
81_1_10	4.698.753	1.032.303	2526	4x	4xA	0
81_1_12	4.698.753	1.032.303	2526	4x	4xA	0
83_3_2	470.407	1.269.105	2271	4x	4xA	0
104_1_3	4.645.142	1.030.158	2298	4x	4xA	0
104_2_1	4.645.142	1.030.158	2298	4x	4xA	0
111_2_1	44.279	6.719.222	2243	4x	4xA	0
111_3_1	44.279	6.719.222	2243	4x	4xA	0
114_2_1	4.472.089	6.919.861	2339	4x	4xA	0
114_2_3	4.472.089	6.919.861	2339	4x	4xA	0
114_3_1	4.472.089	6.919.861	2339	4x	4xA	0
200_2_3	4.418.431	7.603.472	1390	4x	4xA	0
205_1_3	4.423.711	6.704.778	2265	4x	4xA	0

235_2_1	4.416.689	670.763	1930	4x	4xA	0
4_4_2	4.417.528	689.806	2050	4x	4xM	20
17_1_3	4.505.069	6.390.778	2357	4x	4xM	20
17_2_3	4.505.069	6.390.778	2357	4x	4xM	40
17_3_2	4.505.069	6.390.778	2357	4x	4xM	40
17_4_2	4.505.069	6.390.778	2357	4x	4xM	40
20_1_3	4.634.719	7.724.111	2200	4x	4xM	20
34_2_2	4.524.244	6.951.889	2120	4x	4xM	20
36_1_1	4.538.542	7.043.722	2152	4x	4xM	20
36_1_2	4.538.542	7.043.722	2152	4x	4xM	20
36_4_1	4.538.542	7.043.722	2152	4x	4xM	40
36_4_2	4.538.542	7.043.722	2152	4x	4xM	20
37_3_1	4.561.622	7.552.611	2115	4x	4xM	40
37_3_2	4.561.622	7.552.611	2115	4x	4xM	40
40_1_3	460.825	70.125	1860	4x	4xM	40
40_2_1	460.825	70.125	1860	4x	4xM	60
40_2_2	460.825	70.125	1860	4x	4xM	40
45_1_3	4.657.119	8.410.556	2400	4x	4xM	40
47_1_3	4.654.628	9.211.389	2211	4x	4xM	20
48_1_2	4.647.153	9.728.889	2262	4x	4xM	80
53_1_2	4.654.856	1.043.431	2456	4x	4xM	20
54_1_1	4.627.239	1.057.506	2303	4x	4xM	40
54_1_2	4.627.239	1.057.506	2303	4x	4xM	20
54_2_1	4.627.239	1.057.506	2303	4x	4xM	20
54_2_3	4.627.239	1.057.506	2303	4x	4xM	20
54_4_1	4.627.239	1.057.506	2303	4x	4xM	20
54_4_2	4.627.239	1.057.506	2303	4x	4xM	60
58_1_1	4.645.667	1.188.814	2117	4x	4xM	20
58_1_2	4.645.667	1.188.814	2117	4x	4xM	20
58_1_3	4.645.667	1.188.814	2117	4x	4xM	40

58_2_2	4.645.667	1.188.814	2117	4x	4xM	20
58_2_3	4.645.667	1.188.814	2117	4x	4xM	20
59_1_7	4.666.434	1.218.316	2391	4x	4xM	60
73_1_3	4.721.906	1.031.961	2180	4x	4xM	20
75_1_3	4.652.845	9.811.194	2678	4x	4xM	20
77_3_1	4.636.692	7.652.778	2259	4x	4xM	20
79_3_2	4.698.753	1.035.919	2280	4x	4xM	20
79_4_1	4.698.753	1.035.919	2280	4x	4xM	60
81_1_4	4.698.753	1.032.303	2526	4x	4xM	20
81_1_5	4.698.753	1.032.303	2526	4x	4xM	20
83_4_1	470.407	1.269.105	2271	4x	4xM	20
114_3_3	4.472.089	6.919.861	2339	4x	4xM	20
114_4_2	4.472.089	6.919.861	2339	4x	4xM	20
204_2_3	4.415.725	6.729.195	1640	4x	4xM	20
204_3_1	4.415.725	6.729.195	1640	4x	4xM	20
205_4_1	4.423.711	6.704.778	2265	4x	4xM	20

Table S5. Adapter and primer sequences.

Adaptor		Sequence
EcoRI F	5'-	CTCGTAGACTGCGTACC
EcoRI R	5'-	AATTGGTACGCAGTC
MspI/HpaII F	5'-	GATCATGAGTCCTGCT
MspI/HpaII R	5'-	CGAGCAGGACTCATGA

Table S6. RawGeno input parameters and actuating factors, and resulting no. of markers per primer.

Primer				Sequence
Preselective				
EcoRI		5'	-	GACTGCGTACCAATTCA
MspI/HpaII		5'	-	ATCATGAGTCCTGCTCGG
Selective				
EcoRI		5'	-	GACTGCGTACCAATTCAAC
MspI/HpaII 1	5'-	[FAM]	-	ATCATGAGTCCTGCTCGGCTCG
MspI/HpaII 2	5'-	[HEX]	-	ATCATGAGTCCTGCTCGGCTGA
MspI/HpaII 3	5'-	[NED]	-	ATCATGAGTCCTGCTCGGCATA

Table S7. Raw binary data resulted from the RawGeno package. Check the Supplementary excel file.

	**Bin Size**		**Thresholds**		**Estimation**				
**Dye**	*Min*	*Max*	*RFU*	*Repr.*	N_{Bins}	*Repr.*	$Error_{Bonnin}$	*Polym.*	*No.*
Blue	1.5	1.7	200	90	100	85.15	< 0.01	0.77	139
Green	1.6	2.0	250	90	110	77.78	< 0.00	0.76	187
Yellow	1.5	2.0	150	95	108	92.21	< 0.01	0.79	186

Methods S1. Flow cytometric seed screening (all methods and data from [45]):

To quantify the main mode of reproduction, we determined ploidies of both endosperm and embryo per single seed for each individual. Since many tetraploid plants had a poor seed set, as reported previously [45], we had to restrict the sampling to 551 individuals, which formed each a minimum of five well-developed seeds per flower. Five seeds per plant from at least three plants per population were analyzed with a slightly modified FCSS method. Seeds were placed in 2 ml Eppendorf tubes together with two 0.23 cm steel beads (QIAGEN, Hilden, Germany) and ground in a TissueLyser II mill (QIAGEN, Hilden, Germany) with a stroke rate of 30 Hz for 7 seconds. Further preparation was realized using a two-step procedure described by performing (1) a nuclei isolation step with Otto I buffer: 0.1 M citric acid monohydrate, 0.5% v/v Tween 20 (Sigma-Aldrich Munich, Germany), ddH_2O, and (2) a separate staining step with Otto II buffer: 0.4 M Na_2HPO4, ddH_2O and charged with 3 ng/ml 4',6-diamidinophenyl-indole (Sigma-Aldrich, Munich, Germany). Macerated seeds were incubated for 5 minutes with 200 µl ice-cold Otto I buffer. Suspensions were filtered through 40 µm mesh tubes (Partec, Münster, Germany). 800 µl Otto II buffer were then added and incubated for another 15 minutes before analysis. Ploidy levels of all mother plants were determined on fresh leaves from the cultivated plants using the same methods as described above, except for a slightly prolonged grinding time in the TissueLyser (15 seconds).

All analyses were performed on a CyFlow Space flow cytometer (Partec, Münster, Germany). Histograms were taken and analyzed with the supplied FloMAX Software version 2.2.0 (Quantum Analysis GmbH, Münster, Germany). Leaf material of *Zea mays* (CE-777 strain, provided by Doležel J.) and a diploid tested plant of *R. kuepferi* were used as external reference standard to adjust the gain level of the UV LED lamp. All subsequent analyses were conducted with the same parameters.

Peak ranges for embryo (em) and endosperm (es) were set manually in FloMAX and values of DNA content were calculated as Gaussian means. Ratios of es:em ploidies were calculated to determine whether a seed has been produced sexually (3:2 ratio) or via apomixis (3:1, 2.5:1, 2:1 ratio). Interpretation of all plausible pathways for development and fertilization of seeds of *R. kuepferi* have been adopted from the studies by [45, 52], and provided the basis for our classification: A threshold of 1.65 es:em ratio was set to discriminate between sexual (lower values) and asexual (higher values) cases. Those with ratio values between 1.85 and 2.15 were interpreted as autonomous endosperm development since the second peak was always distinct and as high as the endosperm peak in other pathways. Hence, we interpreted it as endosperm peak, and we excluded the possibility that it could represent just a G2 peak of the growing embryo (G2 peaks are usually much smaller than the respective G1 peak, as only few cells are in the respective stage of the cell cycle). Representative flow cytometric histograms are shown in [45]. We categorized every individual as obligate sexual (only sexual seeds), obligate apomictic (only apomictic seeds) or mixed (sexual as well as apomictic seeds = facultative apomixis) by pooling the results of the analyzed seeds, and calculated percentages (see above in Table S1).

Methods S2. DNA Extraction

For standardized DNA preparation and high yields 1 cm^2 leaf material per sample was disrupted using a QIAGEN TissueLyser II (QIAGEN, Hilden, Germany) with 2 steel beads (diameter: 5 mm) in 2 ml Eppendorf tubes, operating at 25 Hz for 2 mins. Isolation of DNA was realized with QIAGEN DNeasy Plant Mini Kit (QIAGEN, Hilden, Germany) using a slightly modified protocol. During lipid cleansing (step 2) a reduced amount of 360 µl AP1 Buffer was used while 40 µl 2.6% polyvinylpyrrolidone (PVP; Carl Roth GmbH & Co. KG, Karlsruhe, Germany) solution was added to attenuate detrimental influence of high polyphenolic compound content in our samples (Healey et al. 2014). Incubation times for lipid cleansing and elution (step 3, step 12 and 13) were prolonged to 30 mins with preheated elution buffer. Elution of isolated DNA (step 12 and 13) was performed with reduced amount of elution buffer (50 µl each) to gain higher concentrations. Isolate quality and quantity was checked on 1.5% agarose gel (Carl Roth GmbH & Co. KG, Karlsruhe, Germany) and NanoDrop 2000 (Thermo Fisher Scientific, Waltham, MA, USA). Only samples without impurities and a DNA content of at least 20 ng/µl were processed further.

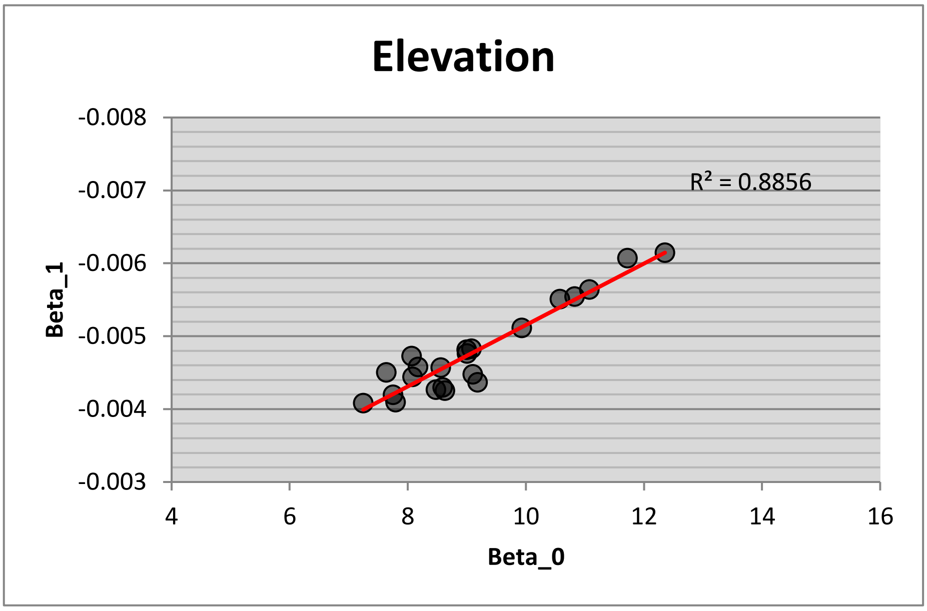

Figure S1. Scatterplot of univariate logistic regression using SamßadaBeta parameters (see Supplementary Table S6) of 22 candidate epilocifor altitude.

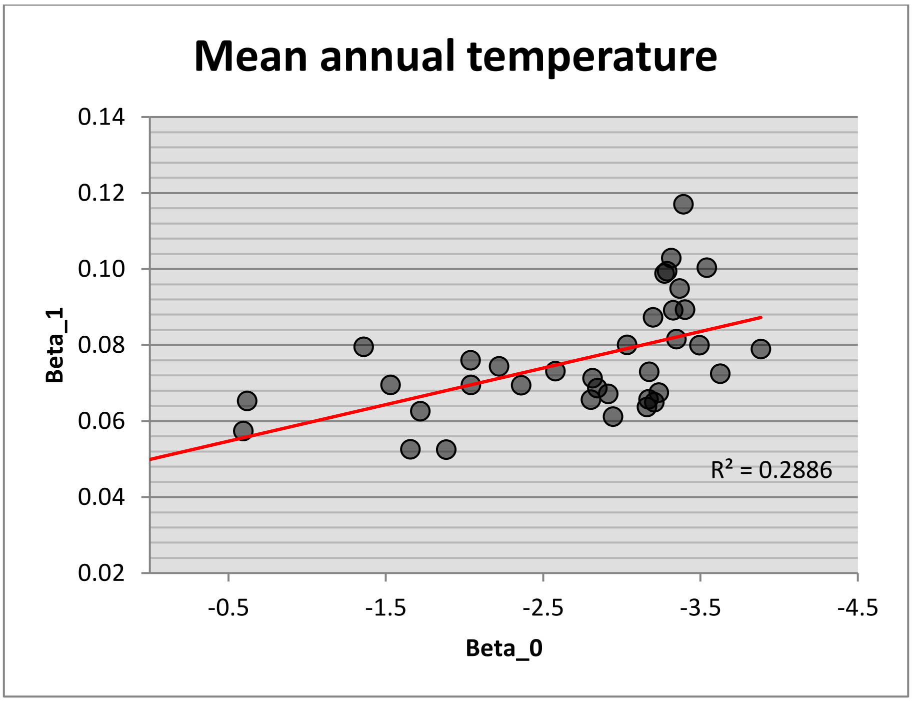

Figure S2. Scatterplot of univariate logistic regression using SamßadaBeta parameters (see Supplementary Table S6) of 36 candidate epilocifor mean annual temperature.

© 2020 by the authors. Submitted for possible open access publication under the terms and conditions of the Creative Commons Attribution (CC BY) license (http://creativecommons.org/licenses/by/4.0/).
